# EGF-loaded, bioactive-rich *Panax notoginseng-*derived nanovesicles accelerate skin wound healing

**DOI:** 10.3389/fcell.2026.1737435

**Published:** 2026-03-12

**Authors:** Fei Yu, Guofeng Xie, Jinfeng Zhao, Xiangsheng Zhang, Zhanxue Xu, Xingyu Lu, Xinrui Yang, Peilin Shi, Tian Li, Yu Zhang, Hongbo Chen

**Affiliations:** 1 School of Pharmaceutical Sciences (Shenzhen), Shenzhen Campus of Sun Yat-sen University, Shenzhen, China; 2 Department of Pharmacy, Chengdu Seventh People’s Hospital (Affiliated Cancer Hospital of Chengdu Medical College), Chengdu, China; 3 Shenzhen Yiai Health Management Co., LTD, Shenzhen, China; 4 School of Chinese Materia Medica, Guangdong Pharmaceutical University, Guangzhou, China; 5 Shenzhen Key Laboratory of Chinese Medicine Active Substance Screening and Translational Research, Department of Pharmacy, The Seventh Affiliated Hospital, Sun Yat-Sen University, Shenzhen, China; 6 Shenzhen Wingor Biotechnology Co., LTD, Shenzhen, China; 7 Department of Dermato-Venereology, The Seventh Affiliated Hospital of Sun Yat-sen University, Shenzhen, China

**Keywords:** cell migration, cell proliferation, epidermal growth factor, Panax notoginseng, plant exosome-like nanovesicles, wound healing

## Abstract

Wound healing is a complex physiological process involving homeostasis, inflammation, proliferation, migration and tissue remodeling. Impaired keratinocyte migration across the wound bed is a key determinant of non-healing wounds. In this context, plant-derived nanovesicles (PDNVs) have emerged as promising therapeutic agents for wound healing due to their high yield, intrinsic biocompatibility, the ability to traverse biological barriers, and an intrinsic molecular cargo (lipids, proteins, nucleic acids, and phytochemicals) that can exert multitarget effects. In this study, we screened a panel of six PDNVs and found *Panax notoginseng*-derived PDNVs (PNVs) displayed superior cell proliferation-promoting activity. To further amplify the bioactivity of PNVs, we actively loaded epidermal growth factor (EGF) onto PNVs (EGF@PNVs). By employing LC-MS and miRNA sequencing, we identified abundant small-molecule compounds (e.g., ginsenoside Rb1, Rg1) and miRNAs (e.g., miRNA 159) in PNVs. *In vitro* experiments demonstrated that PNVs and EGF@PNVs significantly enhanced the proliferation and migration of human keratinocytes (HACAT) as well as the repair of skin mechanical trauma. Moreover, they not only directly accelerated the proliferation and migration of L929 mouse fibroblast cells (L929 cells) but also orchestrated the secretion of TNF-α by mouse mononuclear macrophages (RAW264.7 cells). This cytokine subsequently induced the fibroblast activation or phenotype modulation in L929 cells, further augmenting their proliferative and migratory potential. In a mouse skin injury model both formulations accelerated wound closure and exerted immunomodulatory effects, with EGF@PNVs consistently outperforming PNVs. Collectively, our findings introduce EGF@PNVs as a natural, cost-effective, topical alternative to conventional biologics for wound management.

## 1 Introduction

The skin constitutes the critical physical primary defense shield against pathogen invasion, and its repair involves precisely regulated sequential stages: initial hemostasis, followed by inflammatory response, cellular proliferation, and concluding with tissue maturation (Sun et al., 2014; Park, 2015; Gravitz, 2018; Deniz et al., 2020; Liu et al., 2025). This cascade necessitates coordinated interactions among keratinocytes, fibroblasts, and immune cells, while compromised barrier function increases risks of infection and sepsis (Gurtner et al., 2008; Martin and Nunan, 2015; Amiri et al., 2022). Although animal-derived extracellular vesicles (EVs) participate in wound repair by modulating macrophage polarization and angiogenesis, their clinical translation remains constrained by low yield and high production costs (Han and Ceilley, 2017; Narauskaitė et al., 2021; Al-Masawa et al., 2022; Lin et al., 2022).

Recently, plant-derived nanovesicles (PDNVs), often termed “exosome-like” due to their size (50–300 nm) and cup-shaped morphology, have emerged as promising therapeutic carriers due to their high productivity, low immunogenicity, and inherent drug-loading capacity (Lobb et al., 2015; Doyle and Wang, 2019; Villa et al., 2019; Zhang et al., 2019; Kalluri and LeBleu, 2020). Studies demonstrate that PDNVs from wheat (Şahin et al., 2019), aloe vera (Kim and Park, 2022), and grapefruit (Savcı et al., 2021) accelerate wound healing by enhancing cellular multiplication, motility, and extracellular matrix reorganization. Nevertheless, the reparative mechanisms of PDNVs derived from traditional Chinese medicinal plants remain elusive. Meanwhile, epidermal growth factor (EGF), a classical wound-healing peptide, faces limitations in clinical applications due to enzymatic degradation (Brown et al., 1989; Tsang et al., 2003; Oliveira et al., 2021). The integration of EGF with PDNVs could synergistically combine their advantages, such coordinated efforts are essential to accelerate the responsible integration of EV-based interventions into clinical practice (Verma and Arora, 2025). Yet such combinatorial systems remain unexplored.

This study focuses on *Panax notoginseng*-derived PDNVs (PNVs), employing systematic screening, EGF loading, and multidimensional mechanistic analyses to elucidate their pro-healing effects and signaling network regulation. Our findings aim to advance the development of intelligent plant exosome-based systems for wound regeneration.

## 2 Materials and methods

### 2.1 Cell culture

HaCaT (human keratinocytes), L929 (mouse fibroblasts), and RAW264.7 (mouse mononuclear macrophages) cells were cultured in DMEM medium (Gibco, USA), while Jurkat cells were cultured in RPMI 1640 medium (Gibco, USA). All cell lines were supplemented with 10% fetal bovine serum (FBS; Excell, Australia) and 1% penicillin/streptomycin (Gibco, USA), and maintained at 37 °C in a humidified incubator with 5% CO_2_.

### 2.2 PDNVs isolation

Fresh plants were washed thoroughly, and most of the surface water was allowed to air-dry naturally. The tissue was then minced into small pieces and transferred directly to a blender containing pre-cooled PBS (added at a 1:2 mass ratio). The blender was used to crush the rhizome and extract the juice. The resulting mixture was filtered through gauze to remove large particulate residues, a process repeated once to ensure thorough filtration. PDNVs were extracted from the filtrate using differential centrifugation at the following speeds and durations: 3,000 g for 20 min, 10,000 g for 40 min, 40,000 g for 70 min, and 150,000 g for 90 min. The final precipitate (PDNVs) was resuspended in PBS, filtered through a 0.22 μm membrane, and stored for further use. All steps were conducted on ice or at 4 °C. For long-term storage, the PNVs were stored at −80 °C, while short-term storage at 4 °C was limited to 1 week.

### 2.3 Characterization assay

PNVs and EGF@PNVs were diluted to appropriate concentrations in PBS for characterization. Particle size distribution and zeta potential measurements were employed with a Malvern Zetasizer Pro analyzer. For morphological analysis, 10 µL of the plant vesicle solution was applied to a copper grid and left to stand for 10 min. Excess solution at the edges of the copper grid was gently removed using filter paper. Negative staining involved applying 10 µL of 2% uranyl acetate solution to the grid, followed by a 10-min dark incubation. Excess stain was similarly removed by blotting. The grid was then rinsed with 20 µL PBS for 10 min, and excess liquid blotted again. Finally, the air-dried grid at room temperature was examined using a ZEISS LSM 880 NLO transmission electron microscope. The size distributions and particle concentration of PNVs and EGF@PNVs were measured by nanoparticle tracking analysis (NTA, NS300, Malvern Panalytical). The protein concentration was measured by BCA Protein Assay Kit (Beyotime, P0009) under instructions of the manufacturer. Using the endotoxin detection kit (BOKANG BIO, BK-T04) detect the endotoxin of the protein according to the manufacturer’s instructions.

### 2.4 Epidermal growth factors (EGF) loading and encapsulation efficiency

PNVs solution (1 mg protein, 0.8 mg/mL) was combined with EGF (10 μg, 0.8 μg/mL). And 1 mL aliquot of the mixture was transferred to an electroporation cuvette and subjected to electroporation at 300 V/150 µF. After electroporation, the mixture was left on ice for 1 h to allow membrane recombination, and the electroporation step was repeated. Following centrifugation (15,000 g, 90 min) for supernatant removal, the pellet was resuspended in ice-cold PBS. This washing procedure-resuspension followed by centrifugation-was performed twice to eliminate unencapsulated EGF. The final pellet (EGF@PNVs) underwent 0.22 μm membrane filtration prior to storage. To determine the encapsulation efficiency of EGF in the nanovesicles, the EGF-loaded nanovesicles were disrupted using ultrasonication on ice to release the encapsulated EGF. The EGF content in the nanovesicles was then quantified using an ELISA kit (Elabscience, China). All operations were conducted on ice or at low temperatures. The EGF@PNVs were stored at −80 °C for long-term use or at 4 °C for up to 1 week. For synergy validation experiments, a physical mixture control was prepared by mixed non-encapsulated free EGF with Electroporated PNVs (E-PNVs), incubating at 4 °C for 2 h, and using for cell treatment at equivalent final concentrations as EGF@PNVs. Encapsulation efficiency (EE%) was calculated using the following formula:

$$\text{EE }\left(\%\right)=\frac{\text{Amount}\;\text{of}\;\text{encapsulated}\;\text{EGF}}{\text{Total}\;\text{EGF}\;\text{added}}\times 100\%$$

### 2.5 Epidermal growth factors (EGF) release kinetics assay

EGF@PNVs and control formulations were prepared at equivalent total EGF concentrations (Qtotal). Samples (1 mL) were sealed in dialysis bags (MWCO 14 kDa) and immersed in 100 mL release medium (PBS with 0.1% BSA, 37 °C, 100 rpm shaking). At designated time points (0.5, 1, 2, 4, 8, 12, 24 h), 0.5 mL external medium was collected and replaced with fresh pre-warmed medium. EGF concentration was quantified by ELISA. Cumulative release percentage was calculated as:

$$\text{Cumulative release }\left(\%\right)=\frac{\sum_{i=1}^{n}\left(C_{i}\times V_{i}\right)+C_{n}\times V_{0}}{Q_{\text{total}}}\times 100\%$$

where $C_{i}$ = EGF concentration at time point; $V_{i}$ = sampling volume (0.5 mL); $V_{0}$ = initial medium volume (100 mL), and $Q_{\text{total}}$ = total EGF load in the dialysis bag. The samples were divided into five groups (n = 3 per group): (1) EGF@PNVs; (2) Free EGF; (3) Electroporated PNVs + free EGF mixture; (4) Electroporated PNVs alone (background control); (5) Electroporation buffer (negative control).

### 2.6 Epidermal growth factors (EGF) protease stability assay

EGF@PNVs were washed twice (12,000g, 10 min) to remove unencapsulated EGF, then resuspended in PBS. Samples were incubated with trypsin (final concentration 0.125%) or PBS control at 37 °C. At designated time points (0, 24 h), 50 μL aliquots were withdrawn and immediately mixed with 50 μL Phenylmethylsulfonyl fluoride (PMSF) to terminate proteolysis. Remaining intact EGF was quantified by ELISA and expressed as percentage of initial concentration (0 h):

$$\text{Remaining EGF }\left(\%\right)=\frac{C_{t}}{C_{0}}\times 100\%$$

where $C_{t}$ = EGF concentration at time t, and $C_{0}$ = initial EGF concentration. The samples were divided into five groups (n = 3 per group): (1) EGF@PNVs; (2) Free EGF; (3) Electroporated PNVs + free EGF mixture; (4) Electroporated PNVs alone (background control); (5) Electroporation buffer (negative control).

### 2.7 Cell uptake assay

DIO staining (Beyotime, China) was combined with 1 mL of PBS containing an appropriate amount of PNVs and EGF@PNVs. The mixture was vortexed and dark incubated at 37 °C for 15 min. Post-incubation, the sample was centrifuged (14,000 g, 20 min), the supernatant discarded, and the pellet washed twice with PBS to remove free DIO dye, and the washing step was repeated. The final precipitate was resuspended in PBS, mixed with the culture medium, and used to incubate cells for staining. DIO fluorescence was subsequently observed using laser confocal microscopy and flow cytometry.

### 2.8 Network pharmacological analysis

To identify the potential targets of *P. notoginseng*, the main active components and their related targets were predicted using PharmMapper (http://www.lilab-ecust.cn/pharmmapper/). To screen for targets associated with wound healing, the OMIM (https://www.omim.org/), TTD (https://db.idrblab.net/ttd/), and GeneCards (https://www.genecards.org/) databases were systematically queried using the keyword “wound healing” and all retrieved targets were consolidated. To standardize the predicted targets, all target proteins were mapped to their official gene symbols using the UniProt database (https://www.uniprot.org/), with *Homo sapiens* designated as the target organism. To determine the potential therapeutic targets of *P. notoginseng* in wound healing, the intersection between *P. notoginseng*-related targets and wound healing-associated targets was analyzed using Venny 2.1 software (https://bioinfogp.cnb.csic.es/tools/venny/), with overlapping targets considered as core therapeutic targets mediating the wound healing effects of *P. notoginseng*. The protein-protein interaction (PPI) network was constructed using the STRING database (https://cn.string-db.org/), with *H. sapiens* as the target organism and a minimum interaction score threshold of ≥0.7. Network visualization and topological analysis were subsequently performed in Cytoscape (version 3.9.1) using the CentiScaPe plugin, and the top 15 hub targets were identified based on degree centrality ranking. Gene Ontology (GO) and Kyoto Encyclopedia of Genes and Genomes (KEGG) pathway enrichment analyses of the intersecting targets were conducted using the DAVID database (https://david.ncifcrf.gov/), with *H. sapiens* as the target organism and a false discovery rate (FDR) threshold of <0.05 for significant pathway enrichment. Visualization of the enrichment results was performed using the WeiShengXin platform (https://www.bioinformatics.com.cn/).

### 2.9 LC-MS assay of PNVs

50 µL of the PNVs were diluted with 1 mL methanol solution, and then filtered into sample vial using 0.1 μm filter. The sample extracts were analyzed using an LC-MS system (UPLC, ExionLC AD; MS, QTRAP® 6500+ System, with a UPLC column, Thermo Accucore™ C30 (2.6 μm, 2.1 mm × 100 mm i.d.). The sample was put into the LC-MS sample tray with 2 µL the sample volume and the LC flow rate was 0.5 mL/min (Spray voltage: 4000 V; auxiliary gas: 10 arbitrary unit; sheath gas: 40 arbitrary unit). Mobile phase: pH = 10, H_2_O: MeOH (95:5). The temperature of column is 24 °C. Then, the components in the PNVs were assessed using LC-MS assay.

### 2.10 miRNA sequencing and transfection

Inspired by previous research (Verma et al., 2023), we investigated the miRNA cargo of PNVs. The PNVs were enriched, and 80 µg of PNVs were collected and sent to Lifeint Co (Xiamen, China) for miRNA sequencing, with three biological replicates performed. RNA was extracted using an RNA miRNeasy Micro Kit (Qiagen, Germany), and the quantity and purity were quantified by Quantus Fluorometer (Promega, USA) and Qsep100 (BIOptic, China). Library construction was performed using Multiplex Small RNA Library Prep Set for Illumina (NEB, USA), and subsequent sequencing was performed on the Illumina Novaseq System (Illumina, USA). After aligning the Rfam library to remove ncRNAs (such as rRNA, tRNA, etc.), the reading of each miRNA-seq sample was mapped to specific species precursors in miRbase22.0 to identify known miRNAs. Quantitative statistics of miRNAs were performed using miRDeep2, and differential expression analysis was performed with DESeq2. The mature miRNA target genes were identified using the MultiMiR software package. Exocarpium Citri grandis small RNA miRanda software was used to search cDNA targets of human genes, and default parameter settings were used. The predicted targets of the Exocarpium Citri grandis small RNA were combined with the targets obtained using the multiMiR package, i.e., the predicted target genes. Target genes acquired using the multiMiR package with experimental validation were referred to as validated target genes. Functional enrichment analyses were performed separately for these two target genes. The top 20 miRNAs with the highest read values from the sequencing results were selected for target prediction analysis. The miRNAs mimic oligos were synthesized by Gene Pharma. Cells were transfected with the mimic using GP-transfect-Mate (Gene Pharma) to assess the function of miRNAs on proliferative and macrophage polarization effect.

### 2.11 Cell proliferation

Cell proliferation was assessed via CCK-8 assay (Beyotime, China) measuring OD450 on a microplate reader, and by CFSE staining (Carboxyfluorescein succinimidyl ester, BioLegend, USA) followed by flow cytometric analysis of fluorescence intensity in the FITC channel.

### 2.12 Scratch assay

Cells were plated uniformly in six-well plates (10^6^ cells/mL per well). At full confluence, a cross-shaped scratch was generated perpendicularly across the well using a 200 μL pipette tip, ensuring coverage of upper, lower, left, and right fields. Following washing three times with PBS to clear detached cell debris, the scratch center was microscopically assessed to confirm complete cell removal. Drug treatments were then applied. Scratch images were captured at 0 and 24 h post-treatment, with the wound area quantified and analyzed using ImageJ software.

### 2.13 Conditioned medium collection and fibroblast stimulation

To investigate the indirect effects of PNVs on fibroblasts via macrophage modulation, RAW264.7 cells (mouse) were seeded uniformly into six-well plates and allowed to adhere overnight. After removing the supernatant, RAW264.7 cells were treated with PNVs, following 24 h of induction, the conditioned medium was discarded, and fresh medium was added for an additional 24 h. Then the cell culture supernatant was collected and applied to L929 cells (mouse) to evaluate its effects on the migration and proliferation of L929 cells. This approach ensures species-matched cytokine signaling, as all factors released by RAW264.7 cells act on compatible murine receptors on L929 cells. Meanwhile, L929 cells were treated with or recombinant TNF-α (Novoprotein, CF09) at concentrations matching those measured in the conditioned medium of PNVs-stimulated RAW264.7 cells. Epithelial-to-mesenchymal transition (EMT) marker expression was analyzed by qPCR and Western blot.

### 2.14 Western blot

Total protein was extracted from cells on ice using RIPA lysis buffer (Yeasen Biotechnology, 20120ES60) containing protease inhibitor (Yeasen Biotechnology, 20124ES03), and protein concentration was determined by BCA Protein Assay Kit (Beyotime, P0009). Subsequently, proteins were separated by 10% SDS-PAGE and transferred to PVDF membranes (Millipore). After blocking with TBST containing 5% fat-free dry milk at room temperature for 2 h, the membranes were then separately incubated with the primary antibodies against GAPDH (1:2000, Abmart, P60037), E-cadherin (1:1000, Abmart, TA0131), N-cadherin (1:1000, Abmart, T55015), vimentin (1:1000, Abmart, T55134) at 4 °C overnight. Next, following washing with TBST, the membranes were incubated with HRP-conjugated secondary antibodies (Anti-rabbit IgG, 1:5000, Abmart, B30009) for 1 h. Finally, membranes were washed again with TBST and the protein levels were detected using an enhanced chemiluminescence (ECL) kit (Protein Tech, PK10003).

### 2.15 RNA isolation and qPCR analysis

Cells and ground animal skin tissue samples (processed with a high-throughput freeze grinder) underwent total RNA extraction employing TRIzol® reagent (TaKaRa, Japan). This RNA was then reverse-transcribed to cDNA with the SuperMix Reverse Transcription Kit (TransGen, China). SYBR Green-based qPCR (2 × SYBR Green qPCR Mix, TransGen, China) on a QuantStudio™ five system (Thermo) quantified the cDNA. All steps adhered to the manufacturers’ protocols; primers are listed in Supplementary Table S1.

### 2.16 Mouse skin wound model

All animal experiments were approved by the Institutional Animal Care and Use Committee, Sun Yat-Sen University (Approval number: SYSU-IACUC-2024000236) and conducted following the National standards for animal ethics and protection. The randomization and blinding procedures are described as follows: Mice were assigned to treatment groups using a random number table generated in Microsoft Excel. The allocation sequence was concealed until the moment of treatment administration. To minimize bias, the investigator performing wound photography and area quantification was blinded to group allocation. Treatment applications were performed by a separate investigator. Histological scoring was conducted by two independent, blinded pathologists. The blinding code was broken only after all data had been collected and analyzed. Anesthesia was performed via intraperitoneal injection of pentobarbital (50 mg/kg). The dorsal spine’s lower left side was shaved and depilated. Using an 8-mm skin punch, a full-thickness skin wound extending to the fascial layer was created to ensure evenly sized skin incisions. The mice were randomly divided into five groups (n = 3 per group): Model Group: No treatment; Blank Control Group (PBS): Treated with PBS (5 mL/kg); Empty Vesicle Group (PNVs): Treated with PNVs (5 mg/kg); EGF Group: Treated with EGF (8 μg/kg); Vehicle Group (EGF@PNVs): Treated with EGF@PNVs (5 mg/kg). Treatments were administered topically to the wound site at 0 and 24 h. Body weight changes were recorded daily, and wound sites were photographed every other day using a circular control ring as a reference. Wound healing progression was analyzed using ImageJ software. On day 9, mice were euthanized via cervical dislocation under anesthesia. Wound tissues were harvested for RNA extraction and pathological examination, while spleens were collected for T-cell typing analysis via flow cytometry.

### 2.17 Histology and immunohistochemistry analysis

Skin samples underwent overnight fixation in 4% paraformaldehyde, followed by dehydration in a graded ethanol series, paraffin embedding, and sectioning. Deparaffinized sections were then prepared for hematoxylin and eosin (HE), masson, and immunohistochemical staining. Three wound tissue samples from each group were collected for histopathological observation, which was conducted and photographed using a 10 × microscope.

### 2.18 Statistical analysis

Statistical analyses for all data were performed with at least three independent sample replicates by GraphPad Prism Ver 7.0 software (GraphPad Software Inc., USA). All results are expressed as the mean ± SD. Unpaired Student’s t-test with a two-tailed hypothesis was used to assess the difference between two groups, and one-way analysis of variance (ANOVA) with Tukey’s post-hoc test was applied for multiple groups. The threshold for statistical significance was p < 0.05. Means with different letters differ significantly. Statistical significances between or among the groups were indicated by—ns: no significance; *p < 0.05; **p < 0.01; ***p < 0.001; ****p < 0.0001.

## 3 Results

### 3.1 Preparation and verification of PNVs and EGF@PNVs

**FIGURE 1:**
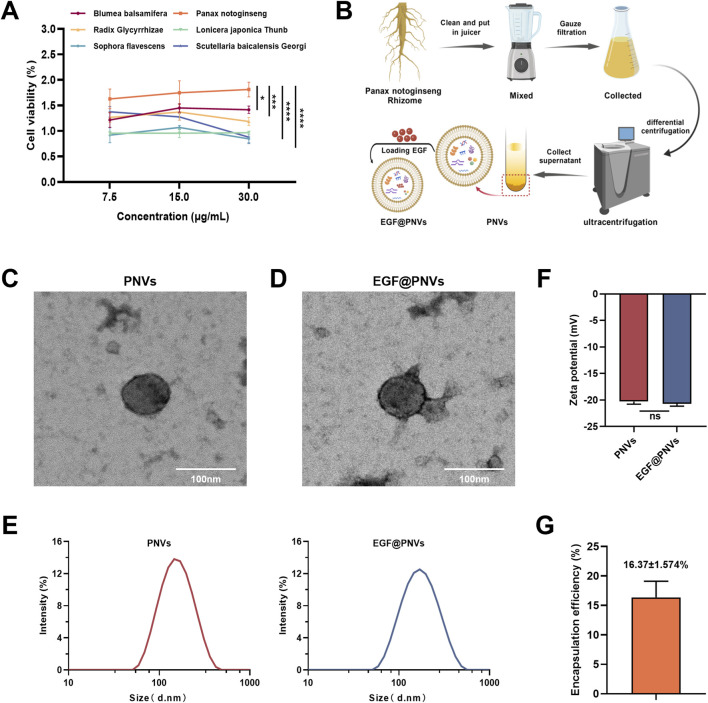
Identification of PNVs and EGF@PNVs. **(A)** Effects of different PDNVs on the proliferation of HaCaT cells (n = 3). **(B)** Schematic illustration showing the extraction process of PNVs and the preparation process of EGF@PNVs. Created with BioGDP.com (Jiang et al., 2025). **(C,D)** Transmission electron microscopy images of PNVs and EGF@PNVs morphology. Scale bar: 100 nm (n = 3). **(E)** Particle size distribution of PNVs and EGF@PNVs (n = 3). **(F)** Typical zeta potential of PNVs and EGF@PNVs (n = 3). **(G)** EGF loading capacity of PNVs (encapsulation efficiency) (n = 3). Data are means ± SD. *p < 0.05, **p < 0.01, ***p < 0.001, ****p < 0.0001, ns represents no significance.

Six medicinal plants with documented dermatological efficacy-*P. notoginseng, Blumea balsamifera, Scutellaria baicalensis, Lonicera japonica, Sophora flavescens, and Glycyrrhiza uralensis*-were selected to screen plant exosome-like nanovesicles (PDNVs) for acute wound therapy. CCK-8 assays revealed that *P. notoginseng*-derived PDNVs (PNVs) exhibited the strongest pro-proliferative activity on HaCaT cells (Figure 1A). Previous studies indicate that *P. notoginseng* components such as ginsenoside Rg1 and Rb1 promote angiogenesis and collagen synthesis by activating FGF-2/PDGFR-β and p38MAPK pathways (Kwok et al., 2012; Zheng et al., 2013; Hou and Kim, 2018; You et al., 2021; Zhang et al., 2021; Lei et al., 2022; Li et al., 2023), justifying the selection of PNVs extracted from *P. notoginseng* rhizomes. PNVs were extracted by differential centrifugation (Figure 1B). Transmission electron microscopy (TEM) confirmed the near-spherical morphology of PNVs (Figure 1C), which remained intact after electroporation-based EGF loading (Figure 1D). Dynamic light scattering (DLS) showed PNVs and EGF@PNVs had a mean particle size of 146.1 nm, with zeta potentials of −20.29 ± 0.2787 mV and −20.7 ± 0.2621 mV, respectively (Figures 1E,F), consistent with plant exosome characteristics (Kim et al., 2022). These physical properties are critical for biological applications: the nanoscale size enables efficient cellular uptake and tissue penetration, while the negative surface charge reduces aggregation and prolongs circulation time. In addition, PNVs, electroporated PNVs (E-PNVs) and EGF@PNVs purified from the same batch were assessed by NTA to measure particle concentration corresponding protein concentrations. The ratio of particle concentration to protein concentration indicated the purification method effectively reduces the residual contamination of miscellaneous proteins (Supplementary Figure S1A, B). We also conducted the Limulus Amebocyte Lysate assay, and the results showed that all samples were below the detection limit (<0.25 EU/mL), confirming that our preparations are endotoxin-free and suitable for *in vivo* applications (Supplementary Figure S1C).

ELISA confirmed an EGF encapsulation efficiency of 16.37% ± 1.574% (Figure 1G), verifying successful EGF@PNVs construction. This moderate loading efficiency is consistent with electroporation-based loading of proteins into plant-derived nanovesicles, where the hydrophilic nature of EGF and its molecular weight (∼6 kDa) limit passive diffusion across the lipid bilayer. Further, we conducted the release experiment of EGF under *in vitro* physiological conditions. Interestingly, we found that the results demonstrate distinct release patterns between different formulations, EGF@PNVs (encapsulated EGF) showed a sustained, gradual release profile characteristic of vesicle-encapsulated cargo. Approximately 15%–20% of total EGF was released at early time points (0–4 h), followed by slow, continuous release reaching ∼30% by 24 h. This biphasic pattern suggested an initial burst release of surface-associated or loosely bound EGF, followed by sustained diffusion from the vesicle interior. Both control (free EGF and E-PNVs + EGF) groups exhibited rapid, complete release within the first 2–4 h, with concentrations plateauing at ∼190–200 pg/mL. This confirms that non-encapsulated EGF is immediately available and does not exhibit sustained release. Electroporated buffer showed negligible EGF signal throughout the study period, confirming that the electroporation buffer itself does not contribute to measured EGF concentrations or introduce artifacts. PNVs alone as expected, no EGF was detected, validating assay specificity (Supplementary Figure S1D). The markedly different release kinetics between EGF@PNVs and free EGF/mix controls provide strong evidence for successful encapsulation rather than simple surface adsorption. We further discovered that Encapsulation within PNVs significantly protects EGF from proteolytic degradation compared to free EGF or physical mixture (Supplementary Figures S1E, F). The incomplete protection (60% degradation) suggested either partial surface-exposed EGF or gradual vesicle permeabilization under protease exposure, consistent with moderate encapsulation efficiency (∼16%) and dynamic release kinetics.

### 3.2 Cellular uptake of PNVs and EGF@PNVs

**FIGURE 2:**
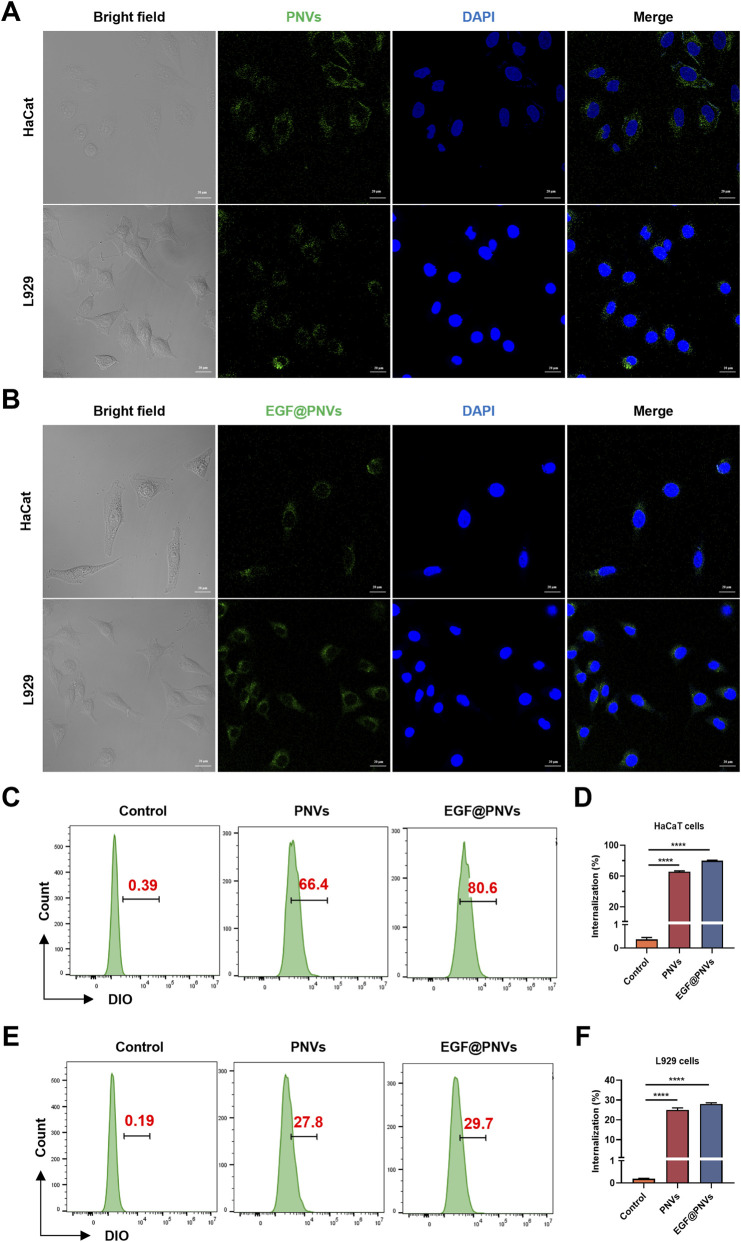
Cells uptake. **(A,B)** Cells were incubated with DIO-labeled PNVs or EGF@PNVs for 24 h for uptake assays. Photographic analysis of DIO and DAPI fluorescence was performed using two-photon laser confocal scanning microscopy to evaluate cell uptake and localization. n = 3 per group. Scale bar: 20 μm. **(C–F)** Representative and quantitative flow cytometry results of the DIO fluorescence signal (n = 3). Data are means ± SD. *p < 0.05, **p < 0.01, ***p < 0.001, ****p < 0.0001, ns represents no significance.

Having established the successful preparation of PNVs and EGF@PNVs, we next investigated their cellular uptake properties. PNVs and EGF@PNVs were labeled by the lipid-structured with DIO (a green fluorescent dye) and counterstained cell nuclei with DAPI (a blue fluorescent dye). Cellular internalization was then analyzed using two-photon laser confocal microscopy. The results revealed that both PNVs and EGF@PNVs localized predominantly around the perinuclear region (Figures 2A,B). Additionally, flow cytometry was employed to quantify the uptake efficiency by measuring DIO fluorescence (FITC channel) in HaCaT and L929 cells after 24 h of incubation with DIO-labeled PNVs and EGF@PNVs (Figures 2C–F).

### 3.3 Mechanistic prediction of PNVs based on small molecule compounds

**FIGURE 3:**
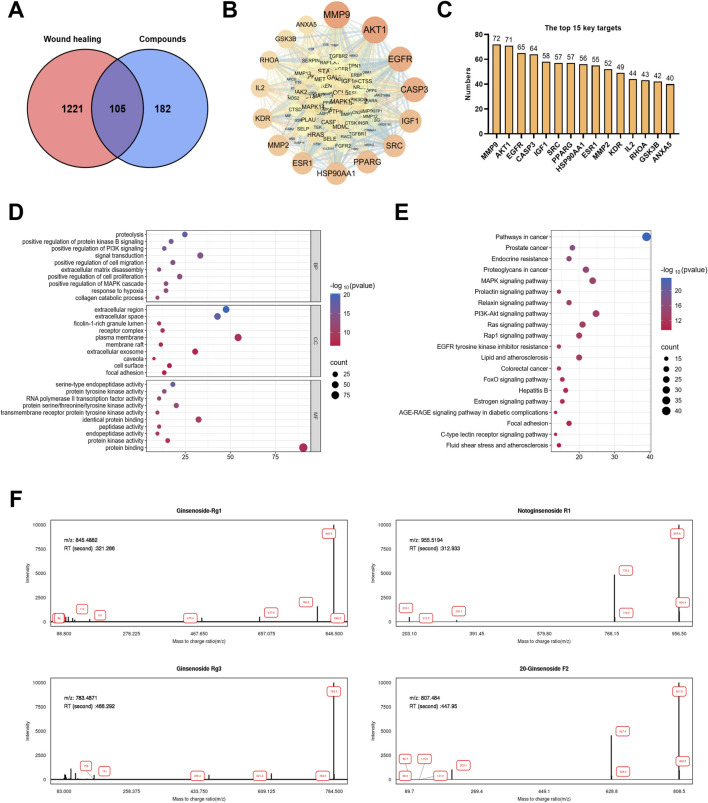
Target prediction analysis of small-molecule compounds by network pharmacology. **(A)** The common targets of wound healing and compounds. **(B)** The PPI network with intersection targets. **(C)** The top 15 key targets. **(D)** GO (Gene Ontology) analysis. **(E)** KEGG (Kyoto Encyclopedia of Genes and Genomes) analysis. **(F)** Verification of the small molecule compounds carried by PNVs using LC-MS (n = 3).

To understand the mechanism of action of PNVs, we next analyzed their bioactive components using network pharmacology. Network pharmacology analysis identified 105 overlapping targets between *P. notoginseng* active components (e.g., ginsenoside Rb1, Rg1) and wound healing-related genes (Figure 3A). In the interaction network, the top 20 potential targets may play an important role (Figures 3B,C). GO enrichment suggested PNVs promote healing by positively regulating cell proliferation, migration, and MAPK/PI3K pathways (Figure 3D). KEGG analysis highlighted significant enrichment in PI3K/AKT and MAPK signaling pathways (Figure 3E), which is particularly significant as these cascades represent convergent signaling nodes regulating cell survival, proliferation, and migration during wound healing. LC-MS confirmed PNVs carried pro-healing small molecules such as ginsenoside Rg1, notoginsenoside R1, ginsenoside Rg3 and ginsenoside F2 (Figure 2F), validating network pharmacology predictions for TCM-derived PDNVs, and these compounds have been independently shown to activate FGF-2/PDGFR-β and p38MAPK pathways in dermal fibroblasts (Kang et al., 2022).

### 3.4 Mechanistic prediction of PNVs based on miRNAs

**FIGURE 4:**
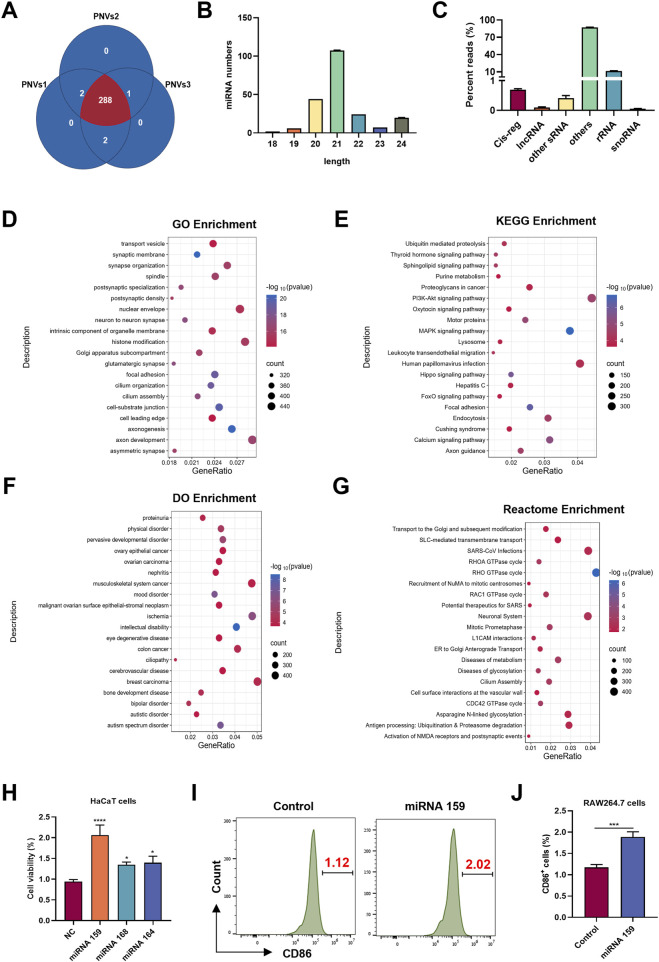
miRNA targets prediction analysis. **(A)** Venn diagram of miRNAs contained in the PNVs (n = 3). **(B)** The length of miRNAs of PNVs (n = 3). **(C)** All mapped clean reads are annotated and the percentage is calculated (n = 3). **(D)** GO enrichment analysis. **(E)** KEGG enrichment analysis. **(F)** DO enrichment analysis. **(G)** Reactome enrichment analysis. **(H)** The top three miRNAs promoted the proliferation of HaCaT cells. **(I,J)** Representative and quantitative flow cytometry results of CD86 expression in RAW264.7 cells by miRNA 159 (n = 3). Data are means ± SD. *p < 0.05, **p < 0.01, ***p < 0.001, ****p < 0.0001, ns represents no significance.

In addition to small molecules, we investigated the miRNA cargo of PNVs. The miRNA sequencing results have been deposited in Figshare (10.6084/m9.figshare.30508319). According to miRNA-sequencing, PNVs from three batches retained an abundance of miRNAs with a length distribution of 20–21 nt, among which 288 candidates were identified (Figures 4A,B). RNA categories, including IncRNA, other sRNA (e.g., miRNA, siRNA), rRNA, and snoRNA, were identified by comparison with mRNA, RFam, and Repbase databases (Figure 4C). miRNA sequencing identified 176 known plant miRNAs in PNVs, the top 20 miRNAs by read counts were enriched in pathways related to proliferation and migration, including PI3K/AKT and focal adhesion (Figures 4D–G). Functional validation showed miRNA 159 exerted the strongest pro-proliferative effect on HaCaT cells (Figure 4H) and upregulated CD86 expression in RAW264.7 cells (Figures 4I,J), suggesting PNVs modulate immune responses via miRNAs. The dominance of miRNA 159 is noteworthy as this miRNA has been evolutionarily conserved across plant species and recently implicated in cross-kingdom gene regulation (Samad et al., 2021). Therefore, we further detected the level of miRNA 159 by transfecting PNVs in HaCaT and RAW264.7 cells. The results indicated that plant miRNA could be delivered to mammalian cells (Supplementary Figures S2A, B).

### 3.5 PNVs promote wound healing by regulating immune cell activity

**FIGURE 5:**
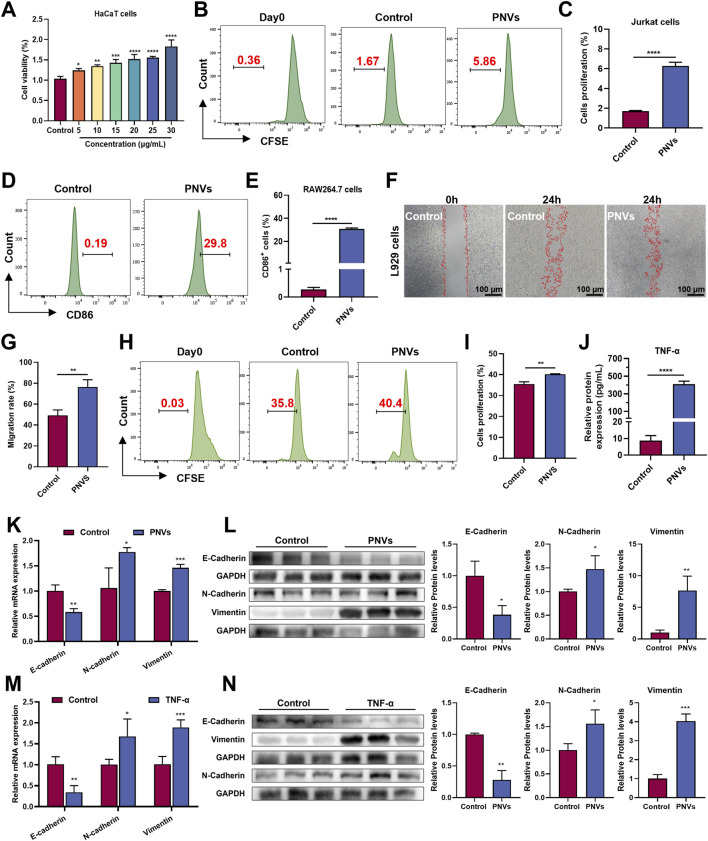
Evaluation of PNVs. **(A)** Concentration-dependent effects of PNVs on HaCaT cell viability (n = 3). **(B,C)** Representative and quantitative flow cytometry results of proliferation stimulation in Jurkat cells (n = 3). **(D,E)** Representative and quantitative flow cytometry results of CD86 expression in RAW264.7 cells by PNVs (n = 3). **(F,G)** Representative and quantitative analysis of cell migration under PNVs treatment as observed in cell scratch assay. Scale bar: 100 μm (n = 3). **(H ,I)** Representative and quantitative flow cytometry results of proliferation stimulation in L929 cells (n = 3). **(J)** The secretion of TNF-α in RAW264.7 cells (n = 3). **(K)** The mRNA expression of EMT-related genes in L929 cells induced by PNVs-CM (n = 3). **(L)** Typical Western blot bands and quantitative analysis of the EMT-related protein levels in L929 cells, induced by PNVs-CM (n = 3). **(M)** The mRNA expression of EMT-related genes in L929 cells induced by TNF-α (n = 4). **(N)** Typical Western blot bands and quantitative analysis of the EMT-related protein levels in L929 cells, induced by TNF-α (n = 3). Data are means ± SD. *p < 0.05, **p < 0.01, ***p < 0.001, ****p < 0.0001, ns represents no significance.

Based on these mechanistic predictions, we next evaluated the functional effects of PNVs on relevant cell types. At a concentration of 30 μg/mL, PNVs dose-dependently enhanced the proliferation of HaCaT keratinocytes (Figure 5A). This pro-proliferative effect aligns with previous reports demonstrating that PDNVs from wheat and grapefruit promote epithelial cell growth through activation of PI3K/AKT and MAPK pathways (Stanly et al., 2020; Zhang et al., 2024). PNVs have also been detected to slightly stimulate the proliferation of Jurkat T cells (Figures 5B,C). Meanwhile, PNVs can also induce M1 polarization in RAW264.7 macrophages (evidenced by increased CD86 expression; Figures 5D,E). Further, we found that conditioned medium derived from PNVs-treated RAW264.7 cells significantly promoted both migration (Figures 5F,G) and proliferation (Figures 5H,I) of L929 fibroblasts. This effect was mediated by elevated TNF-α secretion (Figure 5J) and modulation of epithelial-mesenchymal transition (EMT) markers, characterized by increased expression of *N-cadherin*, *vimentin*, along with decreased *E-cadherin* expression (Figure 5K). Western blot analysis confirmed that PNVs-conditioned medium (PNVs-CM) induced characteristic EMT protein expression changes in L929 fibroblasts: downregulation of epithelial marker E-cadherin and upregulation of mesenchymal markers N-cadherin, vimentin (Figure 5L). Notably, recombinant TNF-α alone (equivalent to PNVs-CM concentration) produced the similar effects with PNVs-CM (Figures 5M,N). We further examined the expression levels of transcription factors *slug* and *ZEB1* and found that PNVs-CM and TNF-α also had significant regulatory effects (Supplementary Figures S3A, B). These findings establish TNF-α as both necessary and sufficient for PNVs-mediated EMT in fibroblasts.

### 3.6 Synergistic pro-healing effects of EGF@PNVs in vitro

**FIGURE 6:**
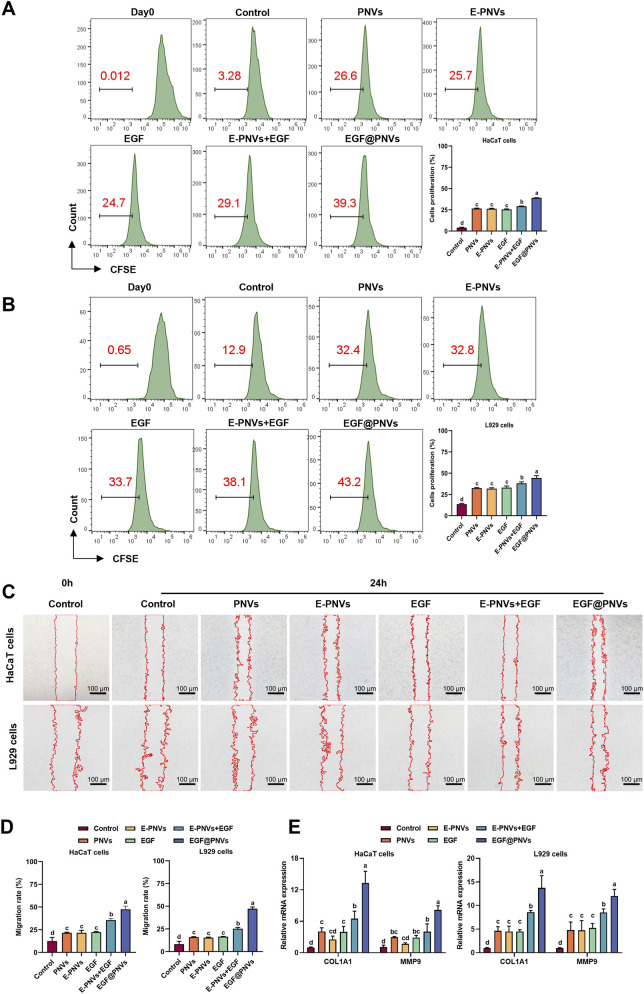
Evaluation of EGF@PNVs *in vitro*. **(A,B)** Representative and quantitative flow cytometry results of cell proliferation of HaCaT cells (top) and L929 cells (bottom) (n = 3). **(C)** Representative flow cytometry results of cell migration of HaCaT cells (top) and L929 cells (bottom) (n = 3). Scale bar: 100 μm. **(D)** Quantitative flow cytometry results of cell migration of HaCaT cells (left) and L929 cells (right) (n = 4). **(E)** The expression of functional genes (*COL1A1* and *MMP9*) (n = 3). Data are means ± SD. The threshold for statistical significance was p < 0.05. Means with different letters differ significantly.

Building upon these findings with PNVs alone, we next examined the synergistic effects of EGF-loaded PNVs. Flow cytometry demonstrated EGF@PNVs significantly enhanced proliferation in HaCaT and L929 cells compared to PNVs or EGF alone (Figures 6A,B). Scratch assays confirmed EGF@PNVs more effectively promoted HaCaT and L929 migration (Figures 6C,D). qPCR revealed EGF@PNVs upregulated *COL1A1* and *MMP9* mRNA (Figure 6E), indicating enhanced extracellular matrix remodeling. Notably, the physical mixture showed only modest enhancement compared to individual treatments, suggesting instability or competitive inhibition when EGF is not encapsulated. In stark contrast, EGF@PNVs achieved significantly superior effects, demonstrating that encapsulation-mediated synergy rather than simple additive effects underlies the enhanced therapeutic efficacy. This finding aligns with reports that extracellular vesicle encapsulation protects growth factors from proteolytic degradation and enables synchronized multi-cargo delivery (Zheng et al., 2023). The superior performance of EGF@PNVs over EGF or PNVs alone or a combination of both demonstrates successful synergistic integration, confirms that the electroporation-based encapsulation process creates a functionally integrated therapeutic system.

### 3.7 Synergistic pro-healing effects of EGF@PNVs in vivo

**FIGURE 7:**
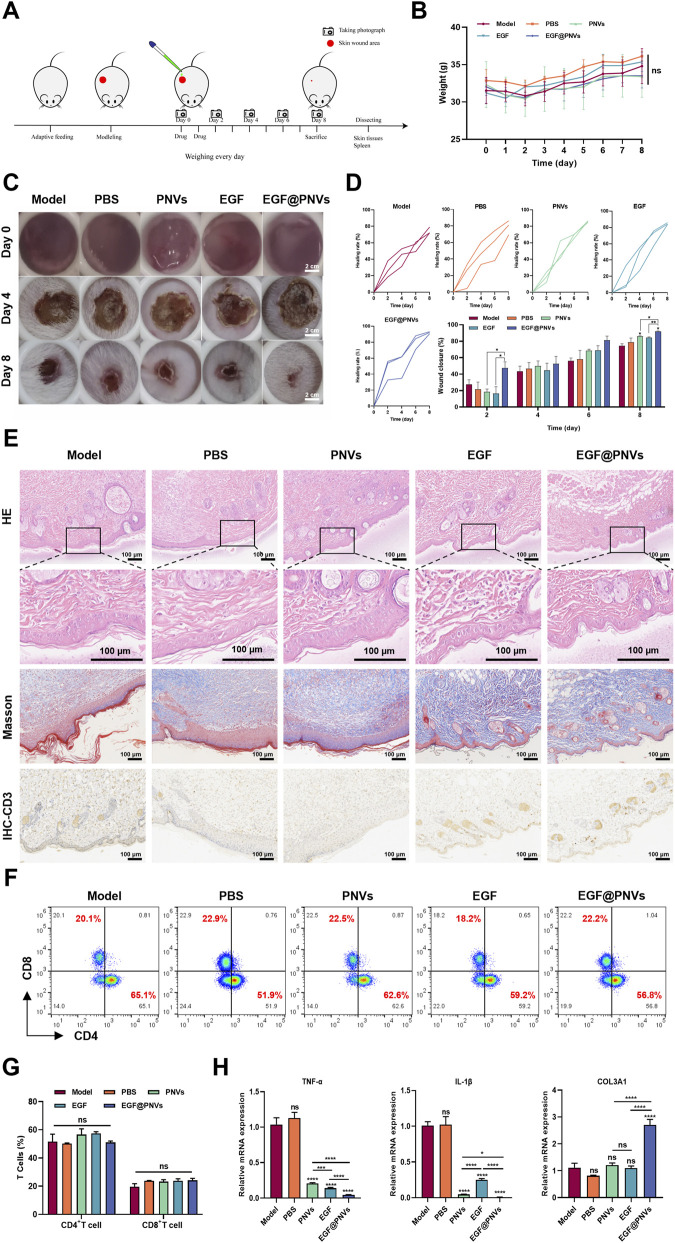
Evaluation of wound healing in mice. **(A)** Schematic diagram of the *in vivo* experimental design. **(B)** Body weight changes recorded daily throughout the 9-day study period (n = 3). **(C)** Representative pictures of wounds in each group on days 0, 4 and 8. A circular reference ring (8 mm diameter) is placed adjacent to each wound as a scale reference (n = 3). Scale bar: 2 cm. **(D)** Quantification of wound closure rates measured every other day from day 0 to day 8 (n = 3). **(E)** Representative H&E staining images showing the wound area at day 9 post-wounding. Low-magnification images display the entire wound region. Higher-magnification images showed the wound center and wound edge regions as indicated. Scale bar: 100 μm (n = 3). Masson trichrome staining of collagen deposition at day 9 post-wounding. Scale bar: 100 μm (n = 3). Immunohistochemical staining of CD3^+^ T-cell infiltration at day 9 post-wounding. Scale bar: 100 μm (n = 3). **(F,G)** Splenic T-cell composition analyzed via flow cytometry on day 9 (n = 3). **(H)** mRNA expression profiles in wound tissues measured by qPCR on day 9 (n = 3). Data are means ± SD. *p < 0.05, **p < 0.01, ***p < 0.001, ****p < 0.0001, ns represents no significance.

Finally, we validated these synergistic effects in an *in vivo* wound healing model. To explore the synergistic pro-healing effects of the EGF@PNVs, we performed the verification experiment in the skin wound models (Figure 7A). No significant weight loss was observed in any treatment group (Figure 7B). In mice with 8-mm full-thickness skin defects, EGF@PNVs achieved a wound closure rate of 91.90% ± 0.7267% by day 8, significantly surpassing PNVs (86.15% ± 0.5359%) and EGF alone (84.54% ± 0.6431%) (Figures 7C,D). Histopathology showed EGF@PNVs-treated wounds exhibited intact epidermal structure, organized collagen deposition, and reduced CD3^+^ T-cell infiltration (Figure 7E). Splenic T-cell subset analysis revealed no significant differences (Figures 7F,G), indicates localized immunomodulation, a safety profile superior to systemic immunosuppressive therapies. The downregulation of TNF-α and IL-1β mRNA at day 9 suggests EGF@PNVs facilitate timely inflammatory resolution, preventing progression to chronic wounds characterized by persistent pro-inflammatory signaling. While EGF@PNVs elevating *COL3A1* expression (Figure 7H), suggesting accelerated healing via anti-inflammatory and pro-matrix synthesis mechanisms.

## 4 Discussion

Plant extracellular vesicles (PDNVs) exhibit unique advantages in wound healing therapy. Current clinical treatments face multiple limitations: small-molecule compounds such as quercetin and curcumin suffer from poor water solubility and instability (Polerà et al., 2019; Kumari et al., 2022), cytokines like EGF and platelet-derived growth factor require careful consideration of formulation stability and transdermal permeability (Steed, 2006; Wei et al., 2022), metal nanomaterials raise biosafety concerns (Servat-Medina et al., 2015; Mehwish et al., 2021; Yang et al., 2021), and mammalian-derived nanovesicles such as mesenchymal stem cell exosomes are hindered by complex extraction processes and high costs (Zhou et al., 2022; Chen et al., 2024). In contrast, PDNVs possess inherent advantages for drug delivery. Their lipid bilayer structure enables stable encapsulation of pharmacologically active components. This study successfully achieved stable EGF loading via electroporation, which not only protects degradable macromolecules like EGF but also circumvents the safety risks of metal nanomaterials. Additionally, their simple production process and low cost make PDNVs highly suitable for large-scale applications. The encapsulation efficiency of 16.37% achieved in our study, while moderate, represents a significant advancement for protein loading into plant-derived nanovesicles. Among them, several factors such as the hydrophilic barrier, the size limitation of EGF (about 6 kDa), and the limitations of electroporation may limit the loading efficiency of EGF in PNVs. Notably, the absolute amount of EGF@PNVs delivered proved therapeutically effective, as evidenced by superior *in vitro* and *in vivo* performance compared to free EGF at equivalent doses. This suggests that loading efficiency alone is not the sole determinant of therapeutic efficacy; rather, the results of the EGF release profile and protease stability experiments confirmed that protection from degradation and sustained release provided by PNVs encapsulation are equally critical factors. While we demonstrated proof-of-concept efficacy with current loading parameters, we acknowledge that further optimization could enhance clinical translation.

Despite the promising preclinical results presented here, several translational hurdles must be addressed before EGF@PNVs can be considered for clinical application. First, scalable and reproducible manufacturing protocols for plant-derived nanovesicles need to be established, including standardized cultivation of source plants, validated isolation methods, and robust quality control metrics (e.g., particle-to-protein ratio, endotoxin levels, batch-to-batch consistency). Second, the stability of PNVs and EGF@PNVs under various storage conditions (lyophilization, room temperature, refrigeration) requires systematic evaluation to enable off-the-shelf availability. Third, topical formulation development (e.g., hydrogels, creams) is needed to ensure adequate retention and penetration at the wound site. Fourth, comprehensive biodistribution and toxicity studies in larger animal models are essential to assess safety. Finally, regulatory pathways for plant-derived nanovesicles are not yet clearly defined, and dialogue with regulatory agencies will be necessary to establish appropriate characterization and quality standards. Addressing these challenges will be critical to advancing EGF@PNVs toward clinical translation.

Network pharmacology analysis predicted that the key therapeutic targets of PDNVs are enriched in MMP9, AKT1, and EGFR. Gene Ontology (GO) functional annotation revealed their significant regulation of cell proliferation, migration, MAPK cascade activation, and PI3K signaling processes. KEGG pathway analysis confirmed PI3K/AKT, MAPK, and Ras signaling as major mechanisms. LC-MS identified PDNVs as natural carriers of wound-healing compounds such as ginsenoside Rg1 and notoginsenoside R1. miRNA sequencing demonstrated that highly abundant miRNAs (e.g., miRNA 159, miRNA 166, miRNA 168) significantly enhance HaCaT cell proliferation. Notably, miRNA 159, the most dominant miRNA, exhibited dual regulatory effects on immune modulation and cell proliferation in transfection experiments, aligning with the holistic therapeutic profile of PDNVs and suggesting a synergistic multi-component mechanism. While our study provides evidence that PNVs deliver miRNA 159 to mammalian cells and that synthetic miRNA 159 mimics recapitulate some of the observed phenotypes, we acknowledge that definitive proof of miRNA 159-mediated cross-kingdom regulation requires (i) antagomir-mediated depletion of miRNA 159 from PNVs to demonstrate loss of function, (ii) identification of direct mRNA targets through transcriptomic analysis combined with miRNA 159 mimic/antagomir treatment, and (iii) validation of candidate targets using 3′UTR luciferase reporter assays. These studies are ongoing in our laboratory and will be reported in future communications.

At the cellular level, PDNVs demonstrate a dual mechanism of action: directly promoting Jurkat cells activation and L929 fibroblast migration, while indirectly modulating the wound microenvironment by stimulating TNF-α secretion and M1 polarization in RAW264.7 macrophages. This “direct action–immune regulation” interplay was validated in a simulated wound microenvironment co-culture system, where conditioned medium experiments showed significantly enhanced L929 cell migration, indicating PDNVs dynamically participate in inflammatory-to-proliferative phase transitions. The demonstration that TNF-α alone recapitulates PNVs-CM effects, provides robust evidence for an indirect, cytokine-mediated mechanism rather than direct vesicle-fibroblast interaction. This finding aligns with the established paradigm of macrophage-fibroblast crosstalk in wound healing, where TNF-α serves as a key paracrine signal coordinating the inflammatory-proliferative phase transition (Peng et al., 2025). However, we acknowledge that TNF-α may not be the sole mediator; other cytokines secreted by PNVs-activated macrophages (e.g., IL-1β, IL-6) may contribute to the observed effects, warranting future cytokine profiling studies. While our data strongly support TNF-α as the primary mediator, we recognize that: (1) neutralizing antibody experiments would definitively establish TNF-α necessity; (2) TNF-α-independent pathways may contribute partially; (3) the reversibility of EMT induction (critical for proper wound resolution) was not assessed in our 24-h assay. These mechanistic nuances represent important directions for follow-up studies.

A limitation of this study is the use of immortalized cell lines (HaCaT, L929, RAW264.7) rather than primary human cells. While the key paracrine signaling axis (RAW264.7 → L929) is species-matched and thus interpretable, the use of human keratinocytes in isolation does not capture potential cross-talk with human immune or stromal cells. Future studies should validate these findings using primary human keratinocytes, fibroblasts, and macrophages, ideally in co-culture or skin equivalent models, to confirm the translational relevance of the observed mechanisms. Additionally, the use of primary human macrophages would allow confirmation of the immunomodulatory effects of PNVs and miRNA 159 in a human context.

EGF-loaded PDNVs (EGF@PNVs) exhibited enhanced efficacy in both *in vitro* and *in vivo* models: *In vitro* studies demonstrated superior proliferative and migratory effects compared to single-component treatments. The inclusion of a physical mixture control group was essential to validate our synergy claims. This distinction has important implications for clinical significance: simply applying EGF and plant exosomes simultaneously (as might occur in traditional herbal medicine combinations) would not achieve the therapeutic efficacy of the engineered EGF@PNVs formulation; *In vivo* animal experiments confirmed accelerated wound closure, with histopathology revealing increased collagen deposition and reduced inflammatory infiltration, without affecting splenic T-cell activity. Importantly, the physicochemical properties of the vesicles remained stable post-loading, underscoring their compatibility as drug carriers. Although the current study employed a minimal number of animals per group (n = 3) in accordance with the 3R principle, we acknowledge that larger cohorts are warranted to confirm the robustness of the observed immunological effects and to enable more detailed subgroup analyses. Future studies will include a pre-determined sample size based on power calculations derived from the effect sizes reported here, and will adhere to strict randomization and blinding protocols.

In normal wound healing, an initial M1 phase is essential for debridement, pathogen clearance, and the release of cytokines (including TNF-α) that recruit and activate fibroblasts and keratinocytes. This is followed by a transition to M2 polarization that promotes resolution and matrix deposition. Our PNV treatment may accelerate or enhance this physiological M1→M2 switch, rather than promoting sustained inflammation. While chronic TNF-α exposure is detrimental, transient TNF-α signaling has been shown to promote fibroblast migration, matrix remodeling, and even re-epithelialization (Barrientos et al., 2008). Our *in vivo* data support this: despite the *in vitro* M1-inducing effect of PNVs, the *in vivo* wounds treated with PNVs or EGF@PNVs showed reduced *TNF-α* and *IL-1β* mRNA at day 9, indicating that the inflammatory response is already resolving by this later time point. The pro-repair effect of M1 signals likely depends on the dose, duration, and microenvironment. Our *in vitro* system captures an acute, transient exposure (24 h conditioned medium), which may mimic the early inflammatory phase of healing.

EMT plays a dual role in wound healing. On one hand, they enable fibroblasts to acquire a migratory phenotype, invade the wound bed, and contribute to granulation tissue formation and matrix remodeling. On the other hand, persistent or dysregulated EMT/fibroblast activation can lead to excessive extracellular matrix deposition, myofibroblast accumulation, and ultimately pathological scarring. In our study, the EMT-like changes observed in L929 fibroblasts exposed to conditioned medium from PNVs-treated macrophages occurred over a relatively short time frame (24–48 h) and were associated with enhanced migration and proliferation. This acute response likely reflects the physiological activation required for wound repair rather than a fibrotic program. Importantly, our *in vivo* data support this interpretation: at day 9 post-wounding, skin tissues from EGF@PNVs-treated mice showed increased collagen deposition but no evidence of disorganized matrix or thickened scar tissue on histological examination. Moreover, the expression of *COL3A1*, a marker of early granulation tissue and favorable remodeling, was significantly upregulated, while inflammatory cytokines (TNF-α, IL-1β) were downregulated, suggesting progression toward a remodeling phase rather than sustained inflammation or fibrosis. However, we acknowledge that our current study was not designed to assess long-term scar outcomes. The 9-day endpoint captures the early-to-mid phases of healing but does not extend to the maturation and remodeling phase (weeks to months in mice), when scar formation becomes fully evident. Therefore, we cannot definitively conclude whether EGF@PNVs treatment ultimately reduces or exacerbates scarring. Notably, the combination of PNVs (which carry anti-inflammatory ginsenosides and immunomodulatory miRNAs) with EGF (which promotes re-epithelialization) may inherently favor a balanced healing response. The transient M1 polarization induced by PNVs may also contribute to effective debridement and pathogen clearance without leading to chronic inflammation, as suggested by the reduced *TNF-α* and *IL-1β* expression at day 9 *in vivo*. Thus, the multi-component nature of EGF@PNVs—combining pro-repair growth factors with anti-inflammatory and immunomodulatory molecules—may help confine the EMT-like activation to the appropriate temporal window, reducing the risk of pathological scarring. This hypothesis, while speculative, provides a foundation for future mechanistic studies.

## 5 Conclusion

**FIGURE 8:**
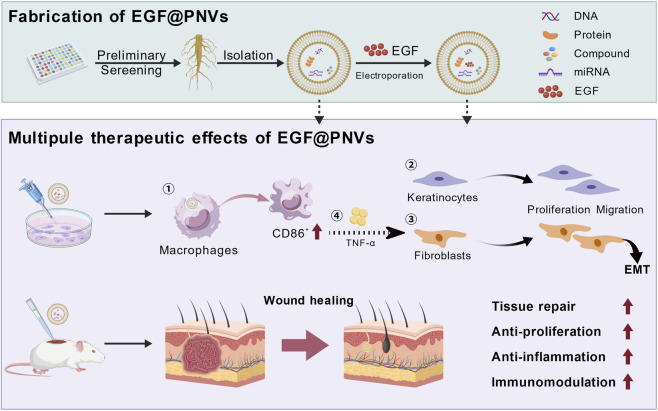
Scheme illustrating EGF@PNVs promoted wound healing. PNVs directly stimulated proliferation and migration in both keratinocytes and fibroblasts. PNVs also regulated macrophage activity and induced fibroblast activation via TNF-α-mediated signaling pathways, indirectly stimulating fibroblast proliferation and migration. This effect may be mediated through the small molecule compounds and miRNAs carried by PNVs. EGF@PNVs exerted the cooperative effect of EGF and PNVs on wound healing. Created with BioGDP.com (Jiang et al., 2025).

EGF@PNVs has a good effect on promoting wound healing both *in vitro* and *in vivo*, and at the later stage of wound healing EGF@PNVs reduces inflammatory infiltration and increases the expression of collagen in the skin wound tissue of mice, and does not affect the activity of T cells in the spleen. EGF loading into PNVs did not change the original properties and physiological activity, and had better pharmacological effects (Figure 8). In addition, PDNVs can be used as drug delivery vehicles, which has broader application prospects compared with animal-derived nanovesicles. Importantly, while PNVs share biophysical properties with mammalian exosomes, they represent a distinct class of plant-derived nanoparticles. Their classification as “exosome-like” is based on operational definitions of size and morphology, not on molecular markers, and this distinction should be considered when interpreting their biological activities.

This study innovatively applied network pharmacology to elucidate the mechanisms of plant-derived vesicles, establishing a new paradigm for traditional Chinese medicine-based nanocarrier research. However, several limitations remain: Current administration methods face challenges in wound retention, necessitating formulation optimizations (e.g., hydrogel-based sustained-release systems) to improve local drug concentration; the dynamic cytokine changes during repair and EMT-related scarring risks require further mechanistic exploration; and comprehensive proteomic/lipidomic profiling of vesicular components is still needed. Future studies should focus on refining production processes, decoding multi-component synergy, and developing clinically relevant chronic wound models to advance the translational potential of plant-derived nanovesicles.

## Data Availability

The datasets presented in this study can be found in online repositories. The names of the repository/repositories and accession number(s) can be found in the article/Supplementary Material.

## References

[B1] Al-Masawa M. E. Alshawsh M. A. Ng C. Y. Ng A. M. H. Foo J. B. Vijakumaran U. (2022). Efficacy and safety of small extracellular vesicle interventions in wound healing and skin regeneration: a systematic review and meta-analysis of animal studies. Theranostics 12 (15), 6455–6508. 10.7150/thno.73436 36185607 PMC9516230

[B2] Amiri N. Golin A. P. Jalili R. B. Ghahary A. (2022). Roles of cutaneous cell-cell communication in wound healing outcome: an emphasis on keratinocyte-fibroblast crosstalk. Exp. Dermatol 31 (4), 475–484. 10.1111/exd.14516 34932841

[B3] Barrientos S. Stojadinovic O. Golinko M. S. Brem H. Tomic-Canic M. (2008). Growth factors and cytokines in wound healing. Wound Repair Regen. 16 (5), 585–601. 10.1111/j.1524-475X.2008.00410.x 19128254

[B4] Brown G. L. Nanney L. B. Griffen J. Cramer A. B. Yancey J. M. Curtsinger L. J. 3rd (1989). Enhancement of wound healing by topical treatment with epidermal growth factor. N. Engl. J. Med. 321 (2), 76–79. 10.1056/nejm198907133210203 2659995

[B5] Chen Z. Zou Y. Sun H. He Y. Ye K. Li Y. (2024). Engineered enucleated mesenchymal stem cells regulating immune microenvironment and promoting wound healing. Adv. Mater 36 (45), e2412253. 10.1002/adma.202412253 39295480

[B6] Deniz A. A. H. Abdik E. A. Abdik H. Aydın S. Şahin F. Taşlı P. N. (2020). Zooming in across the skin: a macro-to-molecular panorama. Adv. Exp. Med. Biol. 1247, 157–200. 10.1007/5584_2019_442 31953808

[B7] Doyle L. M. Wang M. Z. (2019). Overview of extracellular vesicles, their origin, composition, purpose, and methods for exosome isolation and analysis. Cells 8 (7), 727. 10.3390/cells8070727 31311206 PMC6678302

[B8] Gravitz L. (2018). Skin. Nature 563 (7732), S83. 10.1038/d41586-018-07428-4 30464282

[B9] Gurtner G. C. Werner S. Barrandon Y. Longaker M. T. (2008). Wound repair and regeneration. Nature 453 (7193), 314–321. 10.1038/nature07039 18480812

[B10] Han G. Ceilley R. (2017). Chronic wound healing: a review of current management and treatments. Adv. Ther. 34 (3), 599–610. 10.1007/s12325-017-0478-y 28108895 PMC5350204

[B11] Hou J. Kim S. (2018). Possible role of ginsenoside Rb1 in skin wound healing *via* regulating senescent skin dermal fibroblast. Biochem. Biophys. Res. Commun. 499 (2), 381–388. 10.1016/j.bbrc.2018.03.170 29577907

[B12] Jiang S. Li H. Zhang L. Mu W. Zhang Y. Chen T. (2025). Generic Diagramming Platform (GDP): a comprehensive database of high-quality biomedical graphics. Nucleic Acids Res. 53 (D1), D1670–d1676. 10.1093/nar/gkae973 39470721 PMC11701665

[B13] Kalluri R. LeBleu V. S. (2020). The biology, function, and biomedical applications of exosomes. Science 367 (6478), eaau6977. 10.1126/science.aau6977 32029601 PMC7717626

[B14] Kang J. Zhou Y. Zhu C. Ren T. Zhang Y. Xiao L. (2022). Ginsenoside Rg1 mitigates porcine intestinal tight junction disruptions induced by LPS through the p38 MAPK/NLRP3 inflammasome pathway. Toxics 10 (6), 285. 10.3390/toxics10060285 35736894 PMC9228030

[B15] Kim M. Park J. H. (2022). Isolation of Aloe saponaria-Derived extracellular vesicles and investigation of their potential for chronic wound healing. Pharmaceutics 14 (9), 1905. 10.3390/pharmaceutics14091905 36145653 PMC9504946

[B16] Kim J. Li S. Zhang S. Wang J. (2022). Plant-derived exosome-like nanoparticles and their therapeutic activities. Asian J. Pharm. Sci. 17 (1), 53–69. 10.1016/j.ajps.2021.05.006 35261644 PMC8888139

[B17] Kumari A. Raina N. Wahi A. Goh K. W. Sharma P. Nagpal R. (2022). Wound-Healing effects of curcumin and its nanoformulations: a comprehensive review. Pharmaceutics 14 (11), 2288. 10.3390/pharmaceutics14112288 36365107 PMC9698633

[B18] Kwok H. H. Yue P. Y. Mak N. K. Wong R. N. (2012). Ginsenoside Rb_1_ induces type I collagen expression through peroxisome proliferator-activated receptor-delta. Biochem. Pharmacol. 84 (4), 532–539. 10.1016/j.bcp.2012.05.023 22692056

[B19] Lei T. Gao Y. Duan Y. Cui C. Zhang L. Si M. (2022). Panax notoginseng saponins improves healing of high glucose-induced wound through the GSK-3β/β-catenin pathway. Environ. Toxicol. 37 (8), 1867–1877. 10.1002/tox.23533 35385194

[B20] Li D. Wang D. Cai J. Guo Q. Jiang L. (2023). Notoginsenoside R1 facilitates cell angiogenesis by inactivating the notch signaling during wound healing. J. Burn Care Res. 44 (4), 823–831. 10.1093/jbcr/irad035 36905210

[B21] Lin C. W. Hung C. M. Chen W. J. Chen J. C. Huang W. Y. Lu C. S. (2022). New Horizons of macrophage immunomodulation in the healing of diabetic foot ulcers. Pharmaceutics 14 (10), 2065. 10.3390/pharmaceutics14102065 36297499 PMC9606988

[B22] Liu H. Dong T. Dong C. Yang F. Zhou Q. Guan C. (2025). Plant-derived exosome-like nanovesicles: a novel therapeutic perspective for skin diseases. J. Nanobiotechnology 23 (1), 640. 10.1186/s12951-025-03715-1 41074041 PMC12514849

[B23] Lobb R. J. Becker M. Wen S. W. Wong C. S. Wiegmans A. P. Leimgruber A. (2015). Optimized exosome isolation protocol for cell culture supernatant and human plasma. J. Extracell. Vesicles 4, 27031. 10.3402/jev.v4.27031 26194179 PMC4507751

[B24] Martin P. Nunan R. (2015). Cellular and molecular mechanisms of repair in acute and chronic wound healing. Br. J. Dermatol 173 (2), 370–378. 10.1111/bjd.13954 26175283 PMC4671308

[B25] Mehwish H. M. Liu G. Rajoka M. S. R. Cai H. Zhong J. Song X. (2021). Therapeutic potential of Moringa oleifera seed polysaccharide embedded silver nanoparticles in wound healing. Int. J. Biol. Macromol. 184, 144–158. 10.1016/j.ijbiomac.2021.05.202 34089759

[B26] Narauskaitė D. Vydmantaitė G. Rusteikaitė J. Sampath R. Rudaitytė A. Stašytė G. (2021). Extracellular vesicles in skin wound healing. Pharm. (Basel) 14 (8), 811. 10.3390/ph14080811 34451909 PMC8400229

[B27] Oliveira B. C. de Oliveira B. Deutsch G. Pessanha F. S. de Castilho S. R. (2021). Effectiveness of a synthetic human recombinant epidermal growth factor in diabetic patients wound healing: pilot, double-blind, randomized clinical controlled trial. Wound Repair Regen. 29 (6), 920–926. 10.1111/wrr.12969 34563097

[B28] Park K. (2015). Role of micronutrients in skin health and function. Biomol. Ther. Seoul. 23 (3), 207–217. 10.4062/biomolther.2015.003 25995818 PMC4428712

[B29] Peng M. Li N. Wang H. Li Y. Liu H. Luo Y. (2025). Macrophages: subtypes, distribution, polarization, immunomodulatory functions, and therapeutics. MedComm 6 (8), e70304. 10.1002/mco2.70304 40717900 PMC12290311

[B30] Polerà N. Badolato M. Perri F. Carullo G. Aiello F. (2019). Quercetin and its natural sources in wound healing management. Curr. Med. Chem. 26 (31), 5825–5848. 10.2174/0929867325666180713150626 30009700

[B31] Şahin F. Koçak P. Güneş M. Y. Özkan İ. Yıldırım E. Kala E. Y. (2019). *In vitro* wound healing activity of wheat-derived nanovesicles. Appl. Biochem. Biotechnol. 188 (2), 381–394. 10.1007/s12010-018-2913-1 30474796

[B32] Samad A. F. A. Kamaroddin M. F. Sajad M. (2021). Cross-Kingdom regulation by plant microRNAs provides novel Insight into gene regulation. Adv. Nutr. 12 (1), 197–211. 10.1093/advances/nmaa095 32862223 PMC7850022

[B33] Savcı Y. Kırbaş O. K. Bozkurt B. T. Abdik E. A. Taşlı P. N. Şahin F. (2021). Grapefruit-derived extracellular vesicles as a promising cell-free therapeutic tool for wound healing. Food Funct. 12 (11), 5144–5156. 10.1039/d0fo02953j 33977960

[B34] Servat-Medina L. González-Gómez A. Reyes-Ortega F. Sousa I. M. Queiroz Nde C. Zago P. M. (2015). Chitosan-tripolyphosphate nanoparticles as Arrabidaea chica standardized extract carrier: synthesis, characterization, biocompatibility, and antiulcerogenic activity. Int. J. Nanomedicine 10, 3897–3909. 10.2147/ijn.S83705 26089666 PMC4467739

[B35] Stanly C. Alfieri M. Ambrosone A. Leone A. Fiume I. Pocsfalvi G. (2020). Grapefruit-Derived Micro and nanovesicles show distinct metabolome profiles and anticancer activities in the A375 human Melanoma cell line. Cells 9 (12), 2722. 10.3390/cells9122722 33371199 PMC7766354

[B36] Steed D. L. (2006). Clinical evaluation of recombinant human platelet-derived growth factor for the treatment of lower extremity ulcers. Plast. Reconstr. Surg. 117 (7 Suppl. l), 143S–149S. 10.1097/01.prs.0000222526.21512.4c 16799381

[B37] Sun B. K. Siprashvili Z. Khavari P. A. (2014). Advances in skin grafting and treatment of cutaneous wounds. Science 346 (6212), 941–945. 10.1126/science.1253836 25414301

[B38] Tsang M. W. Wong W. K. Hung C. S. Lai K. M. Tang W. Cheung E. Y. (2003). Human epidermal growth factor enhances healing of diabetic foot ulcers. Diabetes Care 26 (6), 1856–1861. 10.2337/diacare.26.6.1856 12766123

[B39] Verma N. Arora S. (2025). Navigating the global regulatory landscape for exosome-based therapeutics: challenges, strategies, and future directions. Pharmaceutics 17 (8), 990. 10.3390/pharmaceutics17080990 40871013 PMC12389065

[B40] Verma N. Khare D. Poe A. J. Amador C. Ghiam S. Fealy A. (2023). MicroRNA and protein cargos of Human limbal epithelial cell-derived exosomes and their regulatory roles in limbal stromal cells of diabetic and non-diabetic corneas. Cells 12 (21), 2524. 10.3390/cells12212524 37947602 PMC10649916

[B41] Villa F. Quarto R. Tasso R. (2019). Extracellular vesicles as natural, safe and efficient drug delivery systems. Pharmaceutics 11 (11), 557. 10.3390/pharmaceutics11110557 31661862 PMC6920944

[B42] Wei Y. Li J. Huang Y. Lei X. Zhang L. Yin M. (2022). The clinical effectiveness and safety of using epidermal growth factor, fibroblast growth factor and granulocyte-macrophage colony stimulating factor as therapeutics in acute skin wound healing: a systematic review and meta-analysis. Burns Trauma 10, tkac002. 10.1093/burnst/tkac002 35265723 PMC8900703

[B43] Yang C. Y. Huang P. H. Tseng C. H. Yen F. L. (2021). Topical Artocarpus communis nanoparticles improved the water solubility and skin permeation of raw A. communis extract, improving its photoprotective effect. Pharmaceutics 13 (9), 1372. 10.3390/pharmaceutics13091372 34575454 PMC8469634

[B44] You S. Shi X. Yu D. Zhao D. An Q. Wang D. (2021). Fermentation of Panax notoginseng root extract polysaccharides attenuates oxidative stress and promotes type I procollagen synthesis in human dermal fibroblast cells. BMC Complement. Med. Ther. 21 (1), 34. 10.1186/s12906-020-03197-8 33446178 PMC7807718

[B45] Zhang Y. Liu Y. Liu H. Tang W. H. (2019). Exosomes: biogenesis, biologic function and clinical potential. Cell Biosci. 9, 19. 10.1186/s13578-019-0282-2 30815248 PMC6377728

[B46] Zhang L. Hu Q. Jin H. Yang Y. Yang Y. Yang R. (2021). Effects of ginsenoside Rb1 on second-degree burn wound healing and FGF-2/PDGF-BB/PDGFR-β pathway modulation. Chin. Med. 16 (1), 45. 10.1186/s13020-021-00455-w 34147112 PMC8214283

[B47] Zhang J. Tian S. Guo L. Zhao H. Mao Z. Miao M. (2024). Chinese herbal medicine-derived extracellular vesicles as novel biotherapeutic tools: present and future. J. Transl. Med. 22 (1), 1059. 10.1186/s12967-024-05892-3 39587576 PMC11587639

[B48] Zheng Y. Feng Z. You C. Jin Y. Hu X. Wang X. (2013). *In vitro* evaluation of Panax notoginseng Rg1 released from collagen/chitosan-gelatin microsphere scaffolds for angiogenesis. Biomed. Eng. Online 12, 134. 10.1186/1475-925x-12-134 24380420 PMC3937171

[B49] Zheng W. Rädler J. Sork H. Niu Z. Roudi S. Bost J. P. (2023). Identification of scaffold proteins for improved endogenous engineering of extracellular vesicles. Nat. Commun. 14 (1), 4734. 10.1038/s41467-023-40453-0 37550290 PMC10406850

[B50] Zhou Y. Zhang X. L. Lu S. T. Zhang N. Y. Zhang H. J. Zhang J. (2022). Human adipose-derived mesenchymal stem cells-derived exosomes encapsulated in pluronic F127 hydrogel promote wound healing and regeneration. Stem Cell Res. Ther. 13 (1), 407. 10.1186/s13287-022-02980-3 35941707 PMC9358082

