# Supplementary material for: EGF-loaded, bioactive-rich Panax notoginseng-derived nanovesicles accelerate skin wound healing
Source: Front Cell Dev Biol. 2026 Mar 12;14:1737435. doi: 10.3389/fcell.2026.1737435 (PMC13019359; doi:10.3389/fcell.2026.1737435)
Supplement: Supplementary file 1 [file DataSheet1.docx]

Supplementary Material

Supplementary Table 1. Primers for quantitative real-time PCR.

Supplementary Figure 1. Characterization of PNVs. (related to Figure 1).

Supplementary Figure 2. The content of miRNA 159. (related to Figure 4).

Supplementary Figure 3. The mRNA expression of EMT-related genes in L929 cells. (related to Figure 5).

**Supplementary Table 1. Primers for quantitative real-time PCR.**

| Gene | Forward primer (5’-3’) | Reverse primer (5’-3’) |
| --- | --- | --- |
| Mouse-β-actin | GGCTGTATTCCCCTCCATCG | CCAGTTGGTAACAATGCCATGT |
| Mouse-TNF-α | CTGAACTTCGGGGTGATCGG | GGCTTGYCACTCGAATTTTGAGA |
| Mouse-IL-1β | GAAATGCCACCTTTTGACAGTG | TGGATGCTCTCAT CAGGACAG |
| Mouse-COL1A1 | AGGGACACAGAGGTTTCAGTGGTT | GCAGCACCAGTAGCACCATCATTT |
| Mouse-COL3A1 | GAGATGTCTGGAAGCCAGAACCATG | ATCTCCCTTGGGGCCTTGAGGT |
| Mouse-MMP9 | TCTACAGAGTCTTTGAGTCCG | GGGCTTCCTCTATGATTCAG |
| Mouse-E-cadherin | CAGGTCTCCTCATGGCTTTGC | CTTCCGAAAAGAAGGCTGTCC |
| Mouse-N-cadherin | AGCGCAGTCTTACCGAAGG | TCGCTGCTTTCATACTGAACTTT |
| Mouse-slug | TGGTCAAGAAACATTTCAACGCC | GGTGAGGATCTCTGGTTTTGGTA |
| Mouse-vimentin | CGTCCACACGCACCTACAG | GGGGGATGAGGAATAGAGGCT |
| Mouse-ZEB1 | GCTGGCAAGACAACGTGAAAG | GCCTCAGGATAAATGACGGC |
| Human-GAPDH | CATGTTCGTCATGGGTGTGAACCA | AGTGATGGCATGGACTGTGGTCAT |
| Human-COL1A1 | AGGGACACAGAGGTTTCAGTGGTT | GCAGCACCAGTAGCACCATCATTT |
| Human-MMP9 | GTGCTGGGCTGCTGCTTTGCTG | GTCGCCCTCAAAGGTTTGGAAT |


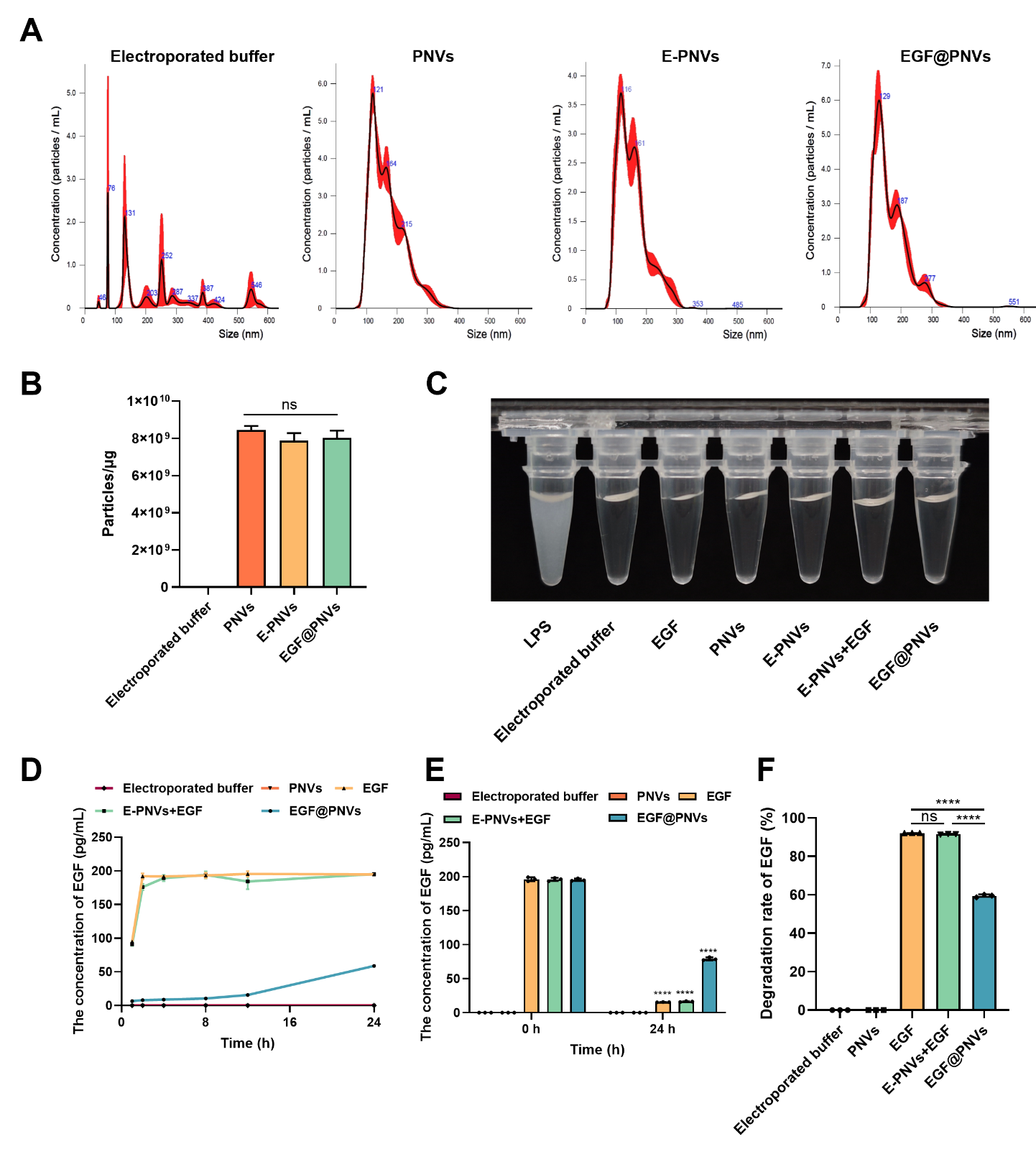


**Supplementary Figure 1.** **Characterization of PNVs.** (A) Representative particle concentration and size distribution of Electroporated buffer, PNVs, E-PNVs and EGF@PNVs by NTA (n=3). (B) The ratio of particle concentration to protein concentration of Electroporated buffer, PNVs, E-PNVs and EGF@PNVs from the same batch at protein concentrations (n=3). (C) Endotoxin detection for different groups of LPS, Electroporated buffer, EGF, PNVs, E-PNVs, E-PNVs+EGF and EGF@PNVs (limit of detection: 0.25 EU/mL) (n=3). (D) EGF release profile at designated time points (0.5, 1, 2, 4, 8, 12, 24 h) (n=3). (E) EGF protease stability assay at designated time points (0, 24 h) (n=3). (F) Degradation rate of EGF (n=3). Data are means ± SD. *p < 0.05, **p < 0.01, ***p < 0.001, ****p < 0.0001, ns represents no significance.


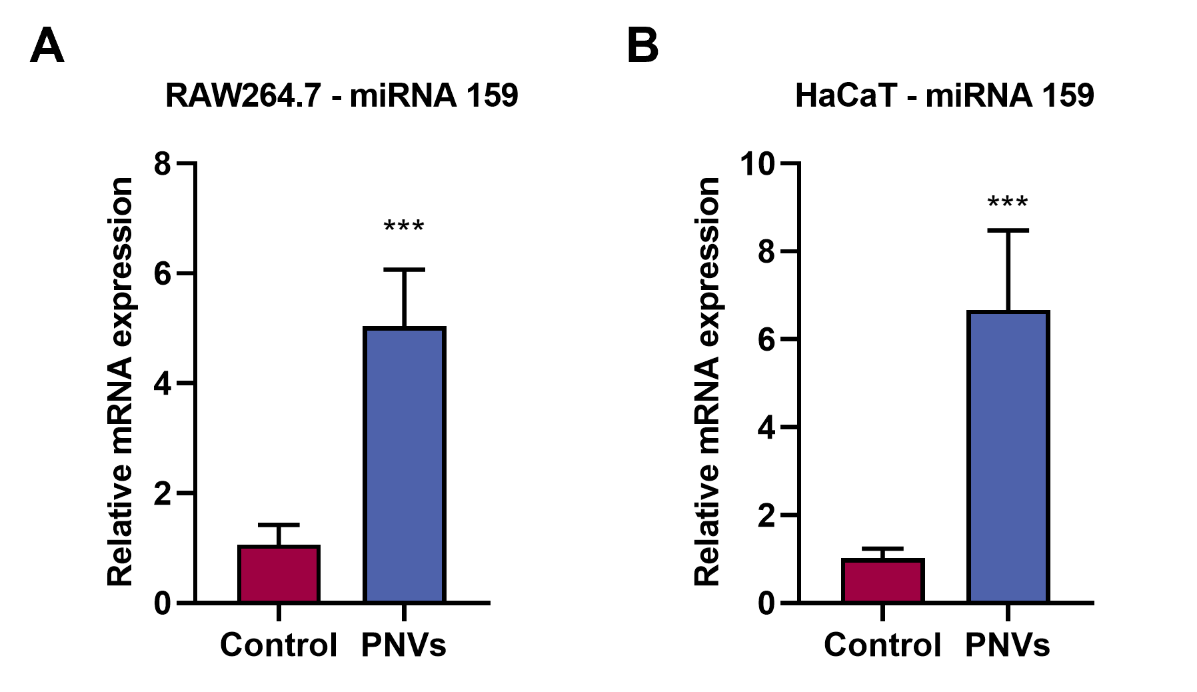


**Supplementary Figure 2. The content of miRNA 159.** (A) The content of miRNA 159 in RAW264.7 cells (n=4). (B) The content of miRNA 159 in HaCaT cells (n=4). Data are means ± SD. *p < 0.05, **p < 0.01, ***p < 0.001, ****p < 0.0001, ns represents no significance.


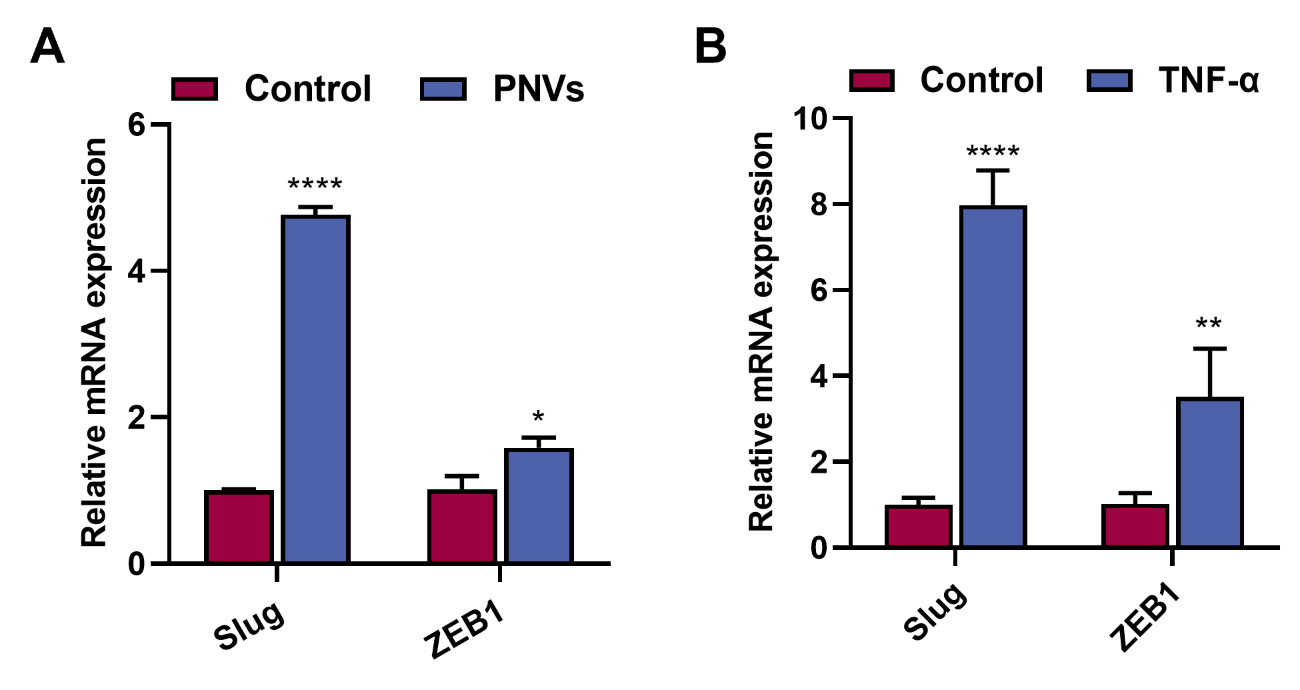


**Supplementary Figure 3**. **The mRNA expression of EMT-related genes in L929 cells.** (A) The mRNA expression of EMT gene in L929 cells by PNVs-conditioned medium (PNVs-CM) (n=3). (B) The mRNA expression of EMT gene in L929 cells by recombinant TNF-α (n=4). Data are means ± SD. *p < 0.05, **p < 0.01, ***p < 0.001, ****p < 0.0001, ns represents no significance.
